# Stress–Strain State Investigation and Ultimate Load on Femoral Implants Based on S-Type Ti6Al4V Titanium Alloy

**DOI:** 10.3390/jfb16050187

**Published:** 2025-05-19

**Authors:** Ivan Panfilov, Ilya Vilkovyskiy, Evgeniy Sadyrin, Sergei Aizikovich, Alexey N. Beskopylny, Besarion Meskhi

**Affiliations:** 1 Department of Theoretical and Applied Mechanics, Agribusiness Faculty, Don State Technical University, Gagarin Square, 344003 Rostov-on-Don, Russia; panfilov.i@gs.donstu.ru (I.P.); saizikovich@gmail.com (S.A.); 2 Department of Veterinary Medicine, RUDN University, 6 Miklukho-Maklaya St., 117198 Moscow, Russia; med-vet@bk.ru; 3 Department of Transport Systems, Faculty of Roads and Transport Systems, Don State Technical University, Gagarin, 1, 344003 Rostov-on-Don, Russia; 4 Department of Life Safety and Environmental Protection, Faculty of Life Safety and Environmental Engineering, Don State Technical University, Gagarin, 1, 344003 Rostov-on-Don, Russia; spu-02@donstu.ru

**Keywords:** endoprosthetics, femoral implant, titanium alloy, finite element analysis, microtomography, reverse engineering, stress concentrators, fracture

## Abstract

Hip replacement is a widespread surgical procedure that eliminates pain and restores motor functions of the pathologically altered hip joint. The issue lies in the lack of pre-operative strength calculations for implant shapes. So, they tend to break after surgery or damage the bone due to the complex stress–strain state. In the present paper, we studied the stress–strain state and ultimate load of S-type canine femoral implants based on titanium alloy Ti6Al4V using finite element analysis for static and cyclic loads. X-ray computed micro tomography data were used to construct the models. Re-engineering and restoration of the 3D geometry of the product were conducted. Strength analysis was performed in the finite element analysis software package Ansys Mechanical was used for various types of implant support. Locations with stress concentrators were identified, and ultimate loads on the implant were obtained. The influence of the rigidity of the support on the prosthesis stem was also studied. For the case of rigid support, the stress–strain state of the prosthesis was studied and the ultimate load was found to be 30.1 kg.

## 1. Introduction

Hip replacement is one of the most common surgical procedures, eliminating pain and restoring patient motor functions with serious pathological alterations of the hip joint, such as osteoarthritis [1,2], after accidents, as well as in older adults. According to the WHO [3], in 2019, the world population aged 60 years and older was just over 1 billion people, which is 13.2% of the total population and 2.5 times more than in 1980 (382 million). It is projected to reach almost 2.1 billion by 2050 [4]. At the same time, age is the strongest predictor of the development and progression of osteoarthritis: approximately 10% of men and 18% of women over 60 years of age suffer from symptomatic osteoarthritis, including moderate and severe forms. In the United States, hip replacement was the fourth most common surgical procedure performed during a hospital stay in 2018 [5,6], and in the European Union, approximately 3.1 million primary hip replacement surgeries have been performed since 1975 [7]. In Russia, from 2008 to 2013, the annual number of large joint replacement surgeries increased over two and a half times, from 33,223 to 86,033, of which 54,703 hip replacements were performed in 2013 [8], while from 2018 to 2020, primary injuries and pathological changes in the hip and femur were registered among over 2.1 million patients [9].

The deformation behavior study of femoral implants for design optimization of medical devices and a more accurate biomimetic correspondence of these artificial structures under the action of physiological loads has been actively developed since the pioneering works [10,11,12,13]. The finite element method (FEM) active development made it possible to model quite complex design options. Thus, Huiskes and Boeklagen [14] presented a numerical shape optimization method, in which FEM is used iteratively to determine the optimal designs of implants that minimize stresses at the interface “surface of the medical device—cement”. Pietrabissa et al. [15] presented a parametric mathematical model of head and cup wear in total hip arthroplasty, while Katoozian and Davy [16] implemented a numerical procedure for three-dimensional optimization of the shape of the femoral component in total hip arthroplasty with the development of an algorithm for determining the geometry of the component in terms of longitudinal and transverse shape variables.

Software and hardware development made it possible to model more realistic load scenarios. Thus, Razumovskii and Shavshukov [17] presented a mathematical model for calculating the stress and strain fields of a hip endoprosthesis design made of a carbon–carbon composite material with a pyrolytic carbon matrix. Maslov et al. [18] performed the stress–strain state finite element analysis of a hip endoprosthesis during walking. Gueiral and Nogueira [19] conducted a study of the stress–strain state for titanium and cobalt–chromium alloys in cementless femoral implants under various loading conditions. Bolshakov et al. [20] developed a computational approach to the structural design of endoprostheses based on Kovin’s principle of bone adaptation to external loads [21]. Ruggiero and Sicilia [22] analyzed the tribological response of an ultra-high-molecular-weight polyethylene artificial hip joint to a ceramic femoral head with changing radial clearance of the implant. Karthik et al. [23] used the FEM to analyze the effect of a porous titanium cup on the stress distribution in a patient’s femur. To minimize stress changes after hip replacement, Naghavi et al. [24] proposed a design of a low-stiffness porous femoral implant made of Ti6Al4V alloy; the stress shielding effect and bone resorption potential of the porous femoral implant were investigated using quasi-physiological experimental analysis and the FEM. Let us note several review works [25,26,27,28,29] on mathematical modeling of the stress–strain state of hip joint implants in various settings.

Currently, the need for femoral implants is also noted for the needs of veterinary medicine. It is assumed that 20% of dogs over 1 year of age suffer from pathological changes in the hip joint, and in some breeds this indicator can be present in over 60% of the population [30], while hip dysplasia occurs in 15.6% of dogs of all breeds in the USA and Canada [31]. Shahar et al. [32] developed an anatomically three-dimensional finite element model of the canine femur loaded with physiological forces, and also constructed the distribution of stresses and deformations in bone tissue, and in [33] studied the distribution of stresses arising in the femur and implant components in two different methods of hip replacement used in clinical practice among dogs. A structural numerical model for optimizing double pelvic osteotomy for early treatment of hip dysplasia among dogs was constructed by Zanetti et al. [34], and a model [35] of the femur–hip contact area was constructed to compare fixation methods in a similar procedure.

Non-destructive testing of implant quality prior to medical intervention is of particular relevance in modern practice due to the likelihood of the presence of artifacts in the internal structure of products. Computed X-ray micro-tomography (micro-CT), which allows obtaining virtual sets of cross-sections of a physical object from any perspective, has proven itself as a tool for defect/porosity analysis [36,37]. This method requires capturing multiple projection images of the sample as it rotates. These projections are then reconstructed into three-dimensional volumes comprising elements (voxels). The entire volume or part of it can be further processed in a digital environment [38,39]. The main difference from conventional computed tomography is the spatial resolution. Compared to computed tomography, where the minimum voxel size is typically <1 mm^3^, the minimum voxel size provided by micro-CT is typically <10 µm^3^ [40,41,42,43,44] which allows the most realistic geometry to be obtained taking into account the manufacturing process, to localize microfractures, cavities, or inclusions. A number of studies show that the geometry of a part manufactured using additive technologies does not always coincide with the micro-geometry from the computer-aided design file used by the operator during the production process [45,46]. Thus, understanding the actual implant geometry becomes critical for good mathematical modeling of the stress–strain behavior of implants [47]. Seemala et al. [48] quantitatively assessed bone density and implant-to-hip contact area in hip arthroplasty using micro-CT and finite element analysis. Similar mathematical and hardware tools were used by Rana et al. [49] to develop an algorithm for bone remodeling during trabecular bone adaptation in the vicinity of a femoral implant. Arachchi et al. [50] and Shim et al. [51] used tomographic scans of hip arthroplasty patients to construct customized FEMs. The use of hip tomographic scans and FEM [52] allowed the loads to be predicted, leading to proximal femur failure. Namvar et al. [53] used micro-CT and FEM to evaluate the mechanical behavior of additively manufactured femoral lattice implants.

Currently, several biocompatible materials are available for hip and knee total-joint replacements; however, titanium alloys (Ti6Al4V [54,55], Ti6Al7Nb [56]) and CoCrMo [57] alloys are the most commonly used. Moreover, modern coatings such as ones based on tantalum [58] including the porous one [59] may significantly improve the bone ingrowth process. Commercially available coatings for implants also include titanium nitride, titanium niobium nitride (Implantcast, Cellumed, OHST medical technology, Link orthopaedics, Corin), oxidized zirconium (Aesculap), and zirconium nitride (Smith and Nephew) coatings [60]. For the advanced implant fixation, especially in the early stage of the bone ingrowth, the bicortical screws may be applied to the implant stem. Among the most promising material for manufacturing such screws are biodegradable alloys based on magnesium [61,62]. These screws can slowly dissolve in the body, disintegrating into safe compounds. This occurs in a controlled manner: the rate of biodegradation should correspond to the gradual replacement of the screw material by bone cells. Thus, the screw will gradually be completely replaced by natural bone.

The aim of this work is to investigate the stress–strain state and ultimate load of the S-type canine femoral implants based on titanium alloy Ti6Al4V using finite element analysis. To build the models, we used micro-CT data obtained by scanning in the vertical stitching mode using additional optical zoom to achieve increased resolution. Re-engineering and restoration of 3D geometry was carried out in the Ansys SpaceClaim geometric editor. Strength analysis was performed in the Ansys Mechanical (v. 2023 R1) finite element analysis software package for different types of supports. Places with stress concentrators were identified, and ultimate loads were obtained. A rigidity effect study of the implant fastening in the femur on the values and places of concentration of maximum stresses was also conducted.

The scientific novelty of the article lies in the development of a mathematical, spatial model of the bone–implant system, numerical analysis of the stress–strain state of the system under various loads and types of boundary conditions, and identification of dangerous zones of potential destruction under static and fatigue effects. It is shown that the elasticity of the fixation (bone rigidity) affects not only the absolute value of the maximum stress (or ultimate load), but also the place of concentration (location of possible destruction). These results allowed clinicians to draw a conclusion about the ultimate loads on the prosthesis (ultimate mass of the animal), to understand the causes of fracture, and revealed the need for further research on the influence of prosthesis fixation.

**Figure 1:**
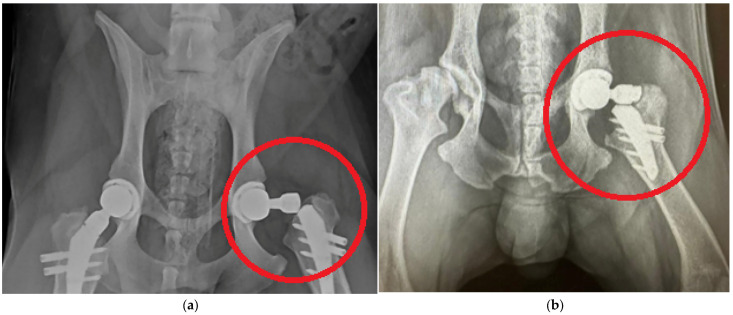
Typical damage to hip endoprostheses during their operation [63]: (**a**) case 1; (**b**) case 2.

Figure 1 shows typical lesions in the upper part of the stem below the neck. Experimental samples of endoprostheses, as well as X-ray research data, were provided by the V@rt company (Moscow, Troitsk, Russia) [63]. The red circle highlights the implant destruction.

## 2. Materials and Methods

**Figure 2:**
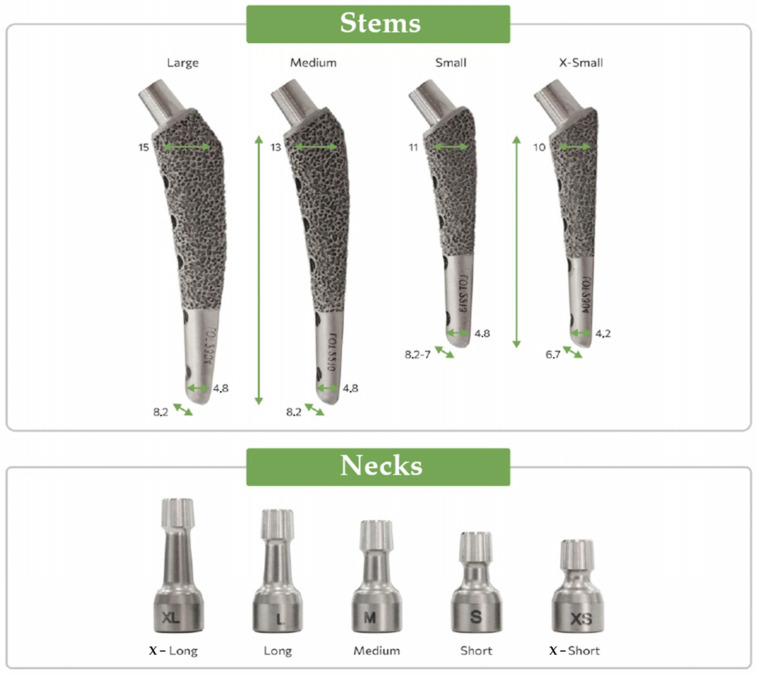
V@rt implants of various sizes [63].

The implant was manufactured using selective laser sintering technology with the subsequent polishing of smooth surfaces. The V@rt canine endoprostheses [63] general view of different sizes is shown in Figure 2.

The part of the endoprosthesis that is directly implanted into the hip is shown—«Stems». The size of the prosthesis is selected based on the size and weight of the dog. A «Neck» is placed on top of the «Stem» during the prosthetic operation, which then interacts with the corresponding part of the pelvic bone (cup and head).

**Figure 3:**
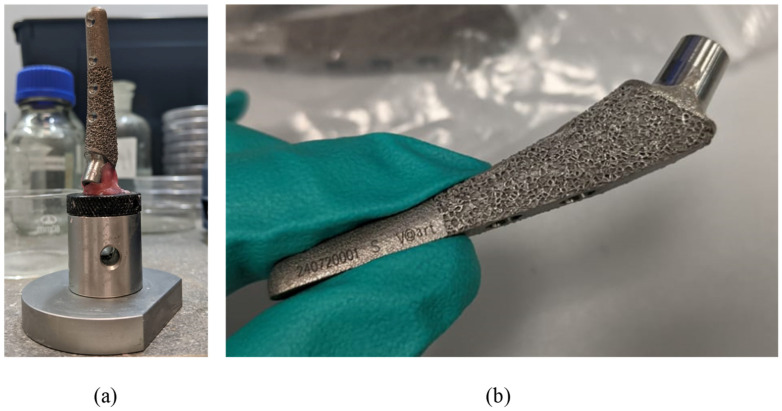
Preparing implants for micro-CT: (**a**) sample fixed on the sample holder; (**b**) sample overview before the fixation procedure.

X-ray images of endoprosthesis fractures in dogs were provided as initial information [63]. Such images allow the general nature of fracture to be understood among operated animals, help to formulate boundary conditions, and also illustrate the relevance of the problem; however, no further in vivo studies are conducted in this paper. Let us note that the prostheses were made of biocompatible Ti6Al4V ELI alloy [64]; the alloy characteristics are given in Table 1 and Table 2. The study used samples of femoral implants of the S size [65] (Figure 3).

The Ti-6Al-4V is an α + β titanium alloy, which is recognized as the most popular titanium alloy used, thanks to the excellent balance of mechanical properties, and lightweight with a density of 4.4 g/cm^3^ [67]. This alloy demonstrates better corrosion resistance than pure medical titanium, due to the surface oxide film that is composed of oxides of alloying elements [68]. Ti-6Al-4V is available in a medically approved material for additive manufacturing in the form of powder. However, when Ti-6Al-4 V alloy is challenged either through corrosion and/or wear particle formation, it may show the cytotoxic effect due to vanadium content [69].

**Figure A1:**
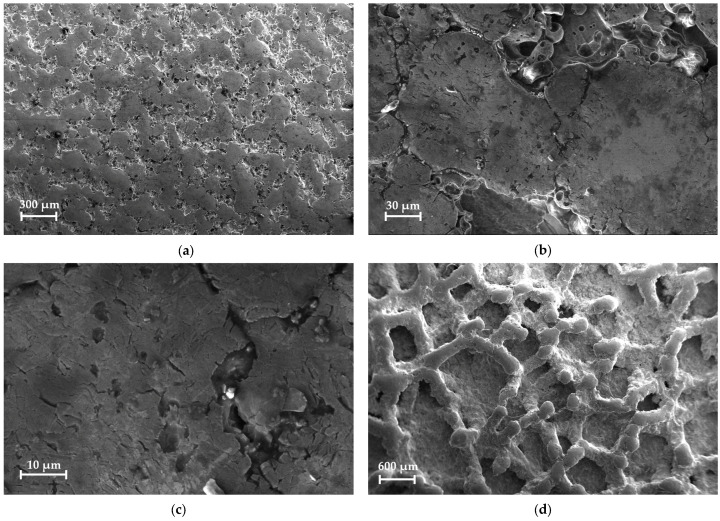
SEM images of the endoprosthesis sample: (**a**) stem smooth surface overview; (**b**,**c**) stem smooth surface microstructure; (**d**) ribbed surface overview.

**Figure A2:**
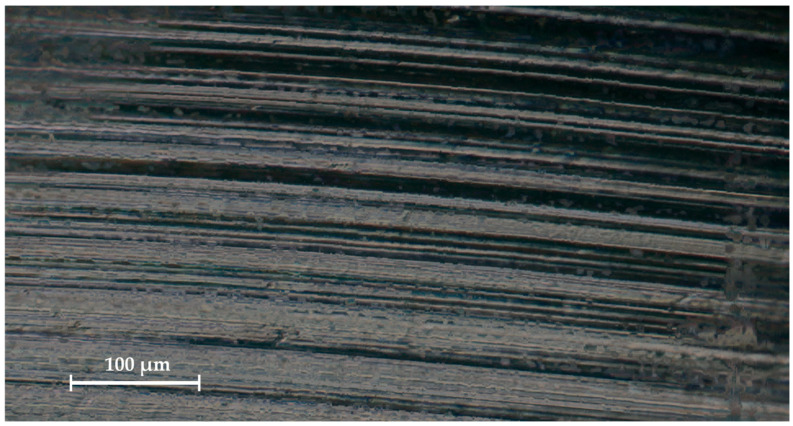
Optical microscope-derived surface of the implant neck.

To review the microgeometric features of the sample, scanning electron microscopy (SEM) of the stem implant surface was performed using an EVO MA18 system (Carl Zeiss Microscopy Deutschland GmbH, Oberkochen, Germany) on smooth (Appendix A, Figure A1a–c) and ribbed (Figure A1c) surfaces. The studies were carried out using an Everhart–Thornley secondary electron detector with an extra high tension of 10 kV. The neck surface was examined using a Zeiss StereoDiscovery V.20 stereomicroscope (Carl Zeiss Microscopy Gmbh, Oberkochen, Germany) according to the Abbe scheme. Zeiss ZEN software (Carl Zeiss Microscopy Gmbh, Oberkochen, Germany) was used for image processing (Appendix A, Figure A2). Significant porosity of about 1 mm is shown in Figure A1d, which corresponds to the ribbed surface on the part of the implant Figure 3b that is placed in the femur and is designed to improve fusion. This ribbing (porosity) is only in the superficial zone, the depth of the ribbing is no more than 1 mm.

### 2.1. Micro-CT Research and Restoration of 3D Geometry

To obtain the digital geometry of the implant, the Xradia Versa 520 micro-CT system (Carl Zeiss Xray Microscopy, Inc., Pleasanton, CA, USA) was used. The scanning parameters are presented in Table 3. To improve the resolution of the final tomogram, scanning was performed with an optical 0.4× magnification in the vertical stitching mode, i.e., for the sample, two scans were performed (the upper and lower segments) separately with a rotation of 360° and 1601 X-ray projections were obtained for each segment, which were then stitched using the Vertical Stitcher (v.11.0.4241.15713) software.

For each scan, the sample was positioned as close as possible to the X-ray source (104.1037 mm from the X-ray tube to the sample rotation axis and 23.9709 mm from the detector to the rotation axis). The 2048 × 2048 pixel CCD camera was maintained at −59 °C and data were collected with a camera binning factor of 2, resulting in projection images up to 1024 × 1024 pixels in size. The optional extra compensatory motions were used to correct for sample drift during scanning. The reconstruction of the projection set into a series of virtual slices with TXM resolution was performed using XRMReconstructor software version 12.0, with the center shift values being defined manually (18.2, 18.6, respectively, for the two tomograms). A Gaussian blur filter (0.5) was applied, and the beam spectrum was shifted to a harder (high-energy) region (by a value of 0.1). A warm-up scan was performed before each scan.

**Figure 4:**
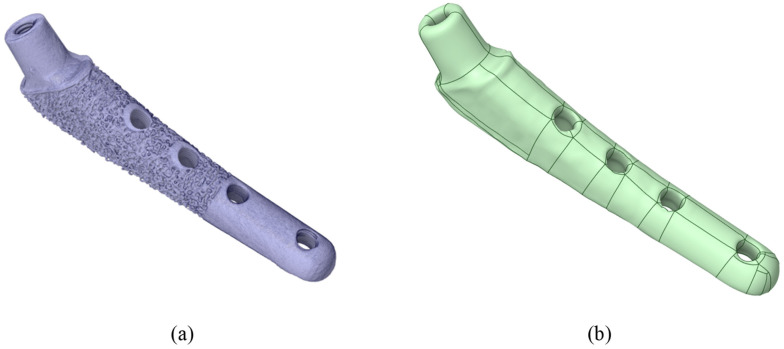
Visualization of the V@RT implant. (**a**) Stl model after microtomography; (**b**) prepared stp model for calculation.

Fiji (v.2.16.0) software was used to convert TXM files to TIFF sequences. VGstudio MAX 3.5 (Volume Graphics GmbH, Heidelberg, Germany) software was used for post-processing of the virtual slice sets of samples, resulting in 3D virtual models of each sample. For 3D visualization, rendering was performed using the Phong shading model [70]. InVesalius 3 software was used to generate STL surfaces. The geometry is shown in Figure 4a.

Then, in the Ansys SpaceClaim (v. 2023 R1) software, the stl surfaces were converted to a vector 3D format, such as stp (Figure 4b).

**Figure 5:**
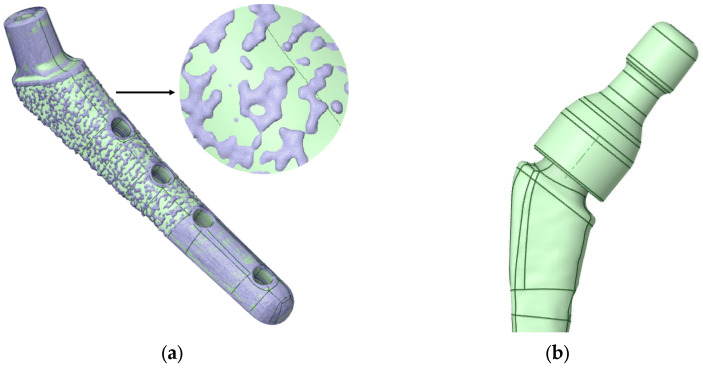
V@RT implants of various sizes. (**a**) Gray—original geometry after scanning in stl format, green—3D geometry prepared for calculation in stp format; (**b**) neck on a stem.

Figure 5a shows the geometry scanned by the micro-CT system and converted for calculation in Gether; as you can see, we have a sufficient match. The ribbed part shown in Figure 5a is intended to improve the fusion of the biological material (bone) with the titanium stem. In this study, uniform adhesion and adhesion of the stem surface immersed in the bone was assumed, so the ribbed part was smoothed (the boundary conditions will be specified in more detail below). However, the influence of the ribbed part on the fusion process and on the occurrence of “micro” stress concentrators are of great interest for further research. Figure 5b shows the neck on the stem.

### 2.2. Numerical Modelling

#### 2.2.1. Core Methods

The main unknowns of the model are stress σ and strain e tensors within the framework of the linear theory of elasticity using the hypothesis of small linear strains [71,72].

To obtain the stress–strain state fields, the numerical FEM [73,74] was used with implementation in the Ansys Mechanical software package [75,76,77].

**Figure 6:**
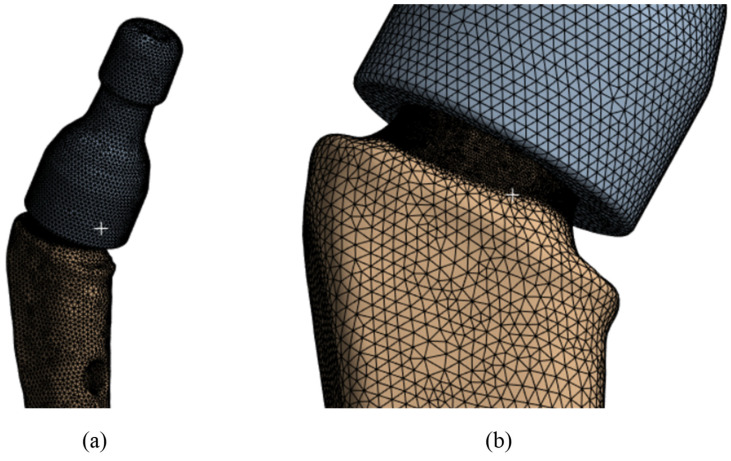
V@RT implants of various sizes: (**a**) the mesh of the elements; (**b**) grid in the proposed concentrator.

The finite element mesh was constructed from tetragonal elements with intermediate nodes of the SOLID187 type in the Ansys Meshing mesh generator with thickening of the area under the neck Figure 6a (in the place of the supposed stress concentrators, Figure 6b). The minimum cell size was taken to be 0.5 mm and in the area below the neck was 0.1 mm. The total number of nodes in this model was 362,780, and the number of elements was 238,616. Verification of the mesh by the number and size of the partition was also conducted, and the results are given below.

A conformal mesh (solid glued material) is made between the neck and the stem, since the neck fits tightly to the stem and no slipping is allowed during operation. In addition, when examining the extracted samples after the fracture [62] of the displacement of the remaining part of the stem, no displacement of the stem inside the neck was detected. Moreover, calculations were performed for contact interaction between the stem and the neck. No effect on the concentrators below the neck was detected.

#### 2.2.2. Strength Criterion

The energy theory (von Mises [78]) was chosen as the first criterion of strength, which assumes the onset of a limiting state in the material when the specific potential energy of shape change in this region reaches the limiting value. The beginning of plastic deformation of the material is chosen as the limiting state.

Thus, the ultimate stresses according to von Mises $\sigma_{M}$ should not exceed the yield stress $\sigma_{T}$ [57,58] of Table 1, i.e., the value of 790 MPa for this alloy.

(1) $$\sigma_{M}\leq \sigma_{T}$$

(2) $$\sigma_{M}=\sqrt{\frac{{\left(\sigma_{xx}-\sigma_{yy}\right)}^{2}+{\left(\sigma_{yy}-\sigma_{zz}\right)}^{2}+{\left(\sigma_{zz}-\sigma_{xx}\right)}^{2}+6{\left(\tau_{xy}^{2}+\tau_{yz}^{2}+\tau_{zx}^{2}\right)}^{2}}{2}}$$

where $\sigma_{xx},\;\sigma_{yy},\;\sigma_{zz}$ are normal stresses, $\tau_{xy},\;\tau_{yz},\;\tau_{zx}$ are tangential stresses.

The second strength criterion is considered to be the fracture due to cyclic loads to which the samples are subjected during movement. In order to avoid using the Wöhler curve to relate the number of cycles and the ultimate stress, we used the theory according to which, to meet the strength criterion for the endurance limit. The ultimate stresses for the alloy should be less than 1/2 of the ultimate stresses [79], i.e., should be less than 430 MPa. In this case, it is assumed that the material withstands the basic number of cycles.

Thus, to meet criteria 1–2, the ultimate stresses $\sigma_{M}$ in the material should not exceed 430 MPa. For static loading, this requirement corresponds to a safety factor of 1.84.

Since the limit stress was set to 430 MPa, which is in the elastic region according to Table 1, a linear elastic model with two parameters (*E* is Young’s modulus and *ν* is Poisson’s ratio according to Table 1) was chosen as the physical model of the material for the finite element calculation. A nonlinear geometric model was used to relate the stress tensor $\varepsilon_{ij}$ and the strain vector $u_{j}$:

(3) $$\varepsilon_{ij}=\frac{1}{2}\left(\frac{\partial u_{i}}{\partial x_{j}}+\frac{\partial u_{j}}{\partial x_{i}}+\frac{\partial u_{i}}{\partial x_{j}}\times \frac{\partial u_{j}}{\partial x_{i}}\right)$$

#### 2.2.3. Loads and Supports

**Figure 7:**
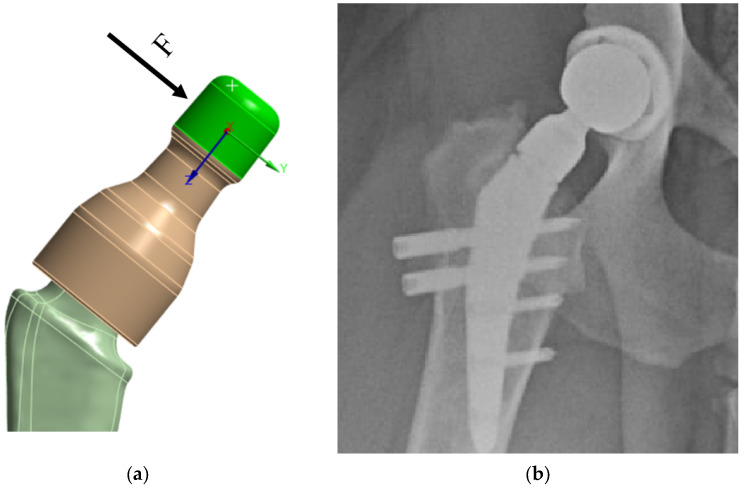
V@rt implants FEM boundary conditions: (**a**) load on the neck; (**b**) X-ray image.

The load from the cup/head is applied as a distributed load along the upper surface of the neck—in green in Figure 7a. Based on the Saint-Venant principle, this application of load is acceptable, since the strength of the prosthesis stem itself is of primary interest, not the neck itself. In this case, preliminary modeling was performed: the load was applied in the OYZ plane based on physiological characteristics, the angle between the main load vector and the Y axis was varied, and the “worst” load direction was chosen. When the load vector is perpendicular to the neck axis, the stresses in the prosthesis reach their limit values, Figure 7a.

**Figure 8:**
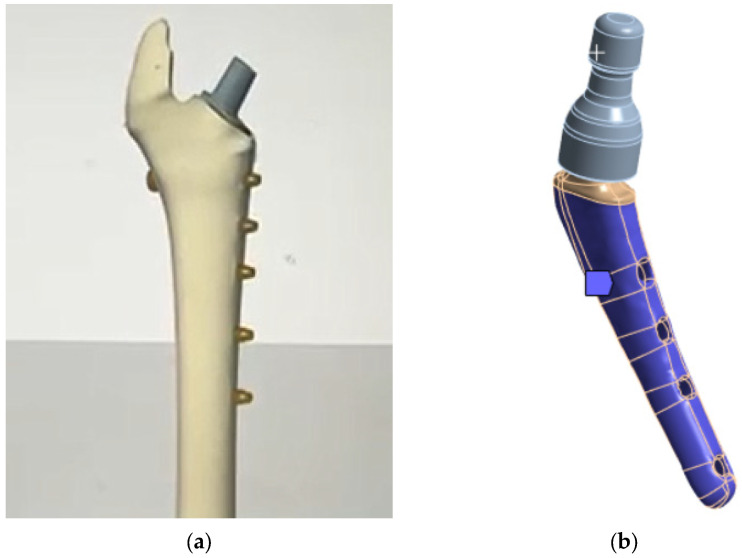
V@RT implants of various sizes: (**a**) bone attachment diagram; (**b**) rigid support.

The lower part of the prosthesis stem is placed in the bone (Figure 7b and Figure 8a). At the moment, there are two technologies for fixing the stem in the bone:

1. Using a special adhesive cement, which, when hardened, provides a strong bond with the bone tissue [80,81];
2. The stem is inserted into the bone tissue without the use of adhesive cement; over time, the bone tissue “grows” around the prosthesis and provides a strong bond. Additionally, fixation with bicortical screws can be used.

Thus, in both cases, a strong adhesion of the prosthesis stem to the bone tissue is implied. In accordance with the initial data and Figure 8b, in the attached model, all degrees of freedom were fixed on the surface in the lower part (in blue)—rigid support (Figure 8b).

## 3. Results and Discussion

**Figure 9:**
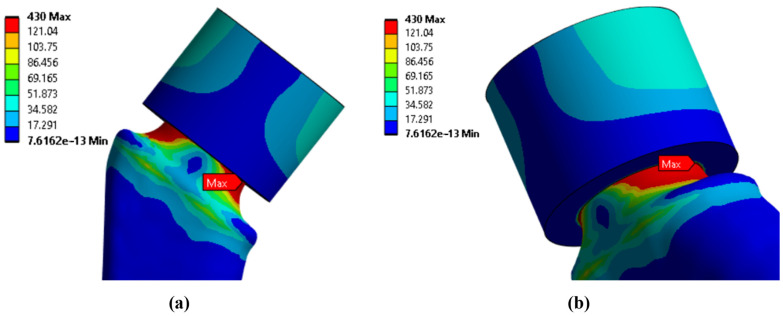
Stress distribution fields according to von Mises, MPa: (**a**) front view; (**b**) side view.

**Figure 10:**
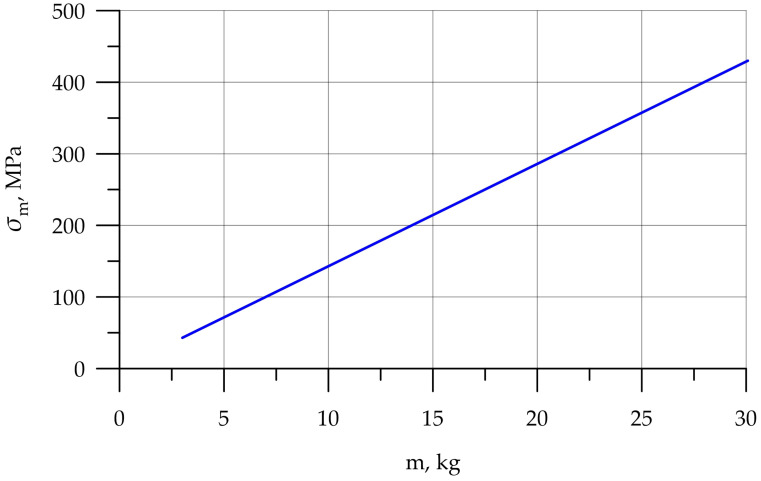
Dependence of stress on applied mass.

**Figure 11:**
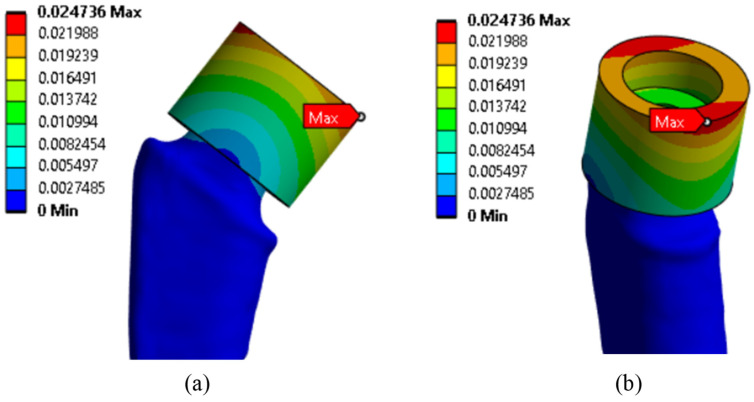
Displacement distribution fields, mm: (**a**) front view; (**b**) side view.

Since the main objective of this work was to study the strength at the fracture sites below the neck, the results for the rigid stem fixation variant are given below. The iterative method was used to select the maximum load on the prosthesis equal to 30.1 kg, at which the maximum von Mises stress value of 430 MPa was achieved, according to the established strength criterion. The stress concentrators were located under the neck. Figure 9 shows the von Mises stress distribution fields, Figure 10 shows a diagram of the dependence of the maximum von Mises stress on the applied mass, and Figure 11 shows the displacements in the model.

The results of stresses were also verified from the dimension of the finite element mesh below the neck (in the locations of the stress concentration); the values of maximum stresses depending on different sizes of the division are presented in Table 4. When the mesh size was reduced to less than 0.1 mm, no significant differences in the results were observed.

**Figure 12:**
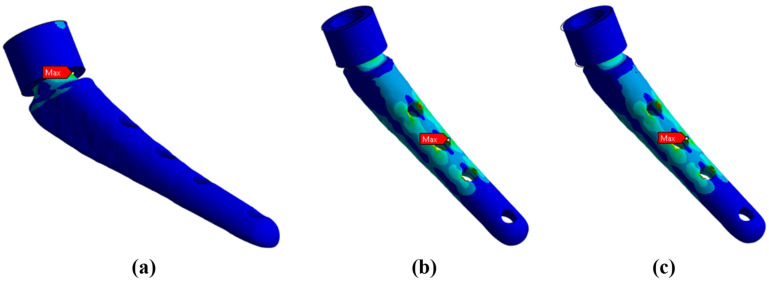
Maximum von Mises stresses for different types of neck support: (**a**) rigid support—218.6 MPa; (**b**) elastic support with the Young’s modulus of 12 GPa—249.1 MPa; (**c**) elastic support with Young’s modulus of 6 GPa—274.9 MPa.

To substantiate the admissibility of the hypothesis of rigid fixation of the lower part of the prosthesis, the results of modeling the support for three cases are presented:

Rigid support of the surface (Figure 12a).

Elastic support of the surface with a rigidity modulus of 12 GPa, which corresponds to the rigidity of bone tissue (Figure 12b).

Elastic support of the surface with a rigidity modulus of 6 GPa, which corresponds to softer bone tissue or insufficiently fused bone tissue (Figure 12c).

Below in Figure 12, the results of the von Mises stress fields for a load of 15 kg are presented. From here on, the upper part of the neck is not shown in the Figures to save space.

**Figure 13:**
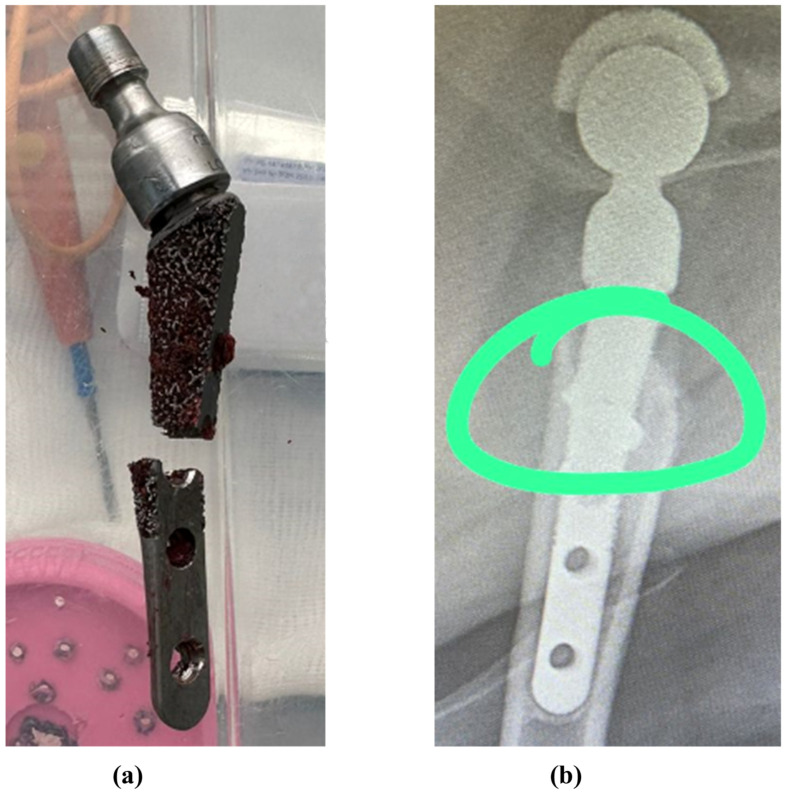
Fractures in the middle part of the stem: (**a**) case 1, (**b**) case 2 [63]. The green circle shows the area of destruction.

It can be noted that the type of support affects not only the absolute values of stress but also the locations of stress concentration. Thus, for fixed support, the stress concentrator, and therefore the fracture site, is located below the neck, which coincides with the radiographs provided in the Figure 1, and also with the results obtained in Figure 9a,b. For elastic support (soft bone tissue or unevenly fused bone tissue), the stress concentrator is located in the middle part of the stem. These types of fracture also occur in Figure 13, but less frequently than the case of fracture below the neck in Figure 2. This fact is quite interesting and requires further detailed study: by varying the support on the stem, it is possible to study, for example, the unevenness of bone tissue ingrowth, the effect of additional bicortical screws, etc.

The results of the work allow the assumption that with a “soft” bone (i.e., insufficiently or unevenly healed), the fracture will be localized in the middle of the stem. In order to avoid the problem of fractures at the early stage of healing, the use of bicortical screws seems extremely appropriate. Moreover, it is possible to use biodegradable screws made of magnesium-based alloys [82,83].

## 4. Conclusions

In this paper, we study the stress–strain state and ultimate load of S-type canine femur implants based on titanium alloy Ti6Al4V ELI using finite element analysis. The following main results are obtained:

1. Using micro-tomography in vertical stitching mode, scanning with additional optical magnification to achieve increased resolution, a three-dimensional image of the implant is reconstructed.
2. Using the re-engineering tools of the Ansys geometric editor, a three-dimensional geometric digital model of the implant is obtained from the micro-tomography data, which is subsequently used in the FEM analysis.
3. Analysis of the stress–strain state of the bone–implant system revealed zones of potential destruction using the finite element method in the Ansys Mechanical software package.
4. The influence of the support rigidity on the prosthesis stem was studied, and it was shown that the support type significantly affects not only the absolute stress values but also the places of their concentration: for an elastic support with a rigidity modulus close to the rigidity modulus of bone tissue, the maximum stress (and, accordingly, the place of a possible fracture) is in the middle of the stem in the area of the holes for the bicortical screws of the endoprosthesis; and for a rigid support, the area of maximum stress values shifts upward under the neck.
5. During the study, places with maximum stress concentrators (according to von Mises) were identified, which coincided with the fracture sites of real samples. This fact may indicate that the destruction of the samples occurred not due to manufacturing inaccuracies (cracks, cavities, or inclusions in the material), but as a result of exceeding the maximum loads on the sample.
6. For the case of a rigid support, the stress–strain state of the prosthesis was studied, and the maximum load was 30.1 kg.

As for the further research, a more detailed study of the boundary conditions on the stress–strain state is planned; in particular, the uneven rigidity of bone tissue and bicortical screws of the endoprosthesis. In addition, a study of the effect of alloys on the stress–strain state, and ultimate loads under static, dynamic, and cyclic load are planned.

In particular, it is planned to conduct a study of prostheses made of medical alloy CoCrMo, and compare the performance characteristics with the alloy Ti6Al4V. It is also planned to use energy-dispersive X-ray spectroscopy to assess the chemical composition of the alloy, X-ray diffraction analysis to assess its phase composition, and nanoindentation to clarify its mechanical characteristics.

## Figures and Tables

**Table 1:** Characteristics of Ti-6Al-4V alloy [64,65,66].

Alloy	Young’s Modulus, GPa	Yield Strength, MPa	Tensile Strength, MPa
Ti-6Al-4V	117	790	860

**Table 2:** Chemical composition Ti-6Al-4V alloy [63,64,65].

Elements, %	V	Al	Fe	O	C	N	Y	Ti
Min.	3.5	5.5	-	-	-	-	-	88.12
Max.	4.5	6.75	0.3	0.2	0.08	0.05	0.005	91

**Table 3:** Parameters of microtomography of the sample.

Tube Voltage, kV	Current, W	Pixel Size, µm	Exposure Time, s	X-Ray Tube Filter
110	9	56	1.0	No

**Table 4:** Verification of stress results from finite element mesh dimensions.

	Mesh 0.5 mm	Mesh 0.1 mm (Calculation Case)	Mesh 0.05 mm	Mesh 0.03 mm
Maximum values of von Mises stress, MPa	425.4	430.0	430.2	430.3
Maximum displacement, mm	0.0246	0.0247	0.0247	0.0247

## Data Availability

The original contributions presented in the study are included in the article; further inquiries can be directed to the corresponding authors.

## Abbreviations

The following abbreviations are used in this manuscript:

Micro-CT	X-Ray computed microtomography
FEM	Finite element method
